# Report of Three Cases in Which Lactation Was Reproduced by the Application of the Child to the Breast

**Published:** 1852-05

**Authors:** Ariel Ballou


From the American Journal of Medical Science.

*Report of three Cases in which Lactation was reproduced by the application of the Child to the Breast.* By Ariel Ballou, M. D. (Read to the Rhode Island Medical Society.)

Case I. In the autumn of 1836, Mrs. J. G., aged between thirty and forty years, of sanguine temperament, robust constitution, and the mother of several children, was confined. The presentation was natural, and no unusual circumstances attended her delivery. Subsequently she suffered from an attack of phlegmasia dolens in both of the lower extremities, attended with high febrile action, and, as is usual in such cases, extreme suffering. The secretion of milk ceasing early in the disease, the child was removed to a wet nurse, with whom it remained three or four months, during which time there was no return of milk. In the spring of 1837, the family being about to move a short distance from the village, where they could enjoy a better air and a more unrestricted exercise, the mother was anxious to take her infant with her, but did not like to deprive it of the advantages of the breast during the then coming warm season. I advised the mother to take her child and apply it to the breasts in the same manner she would do if she had a flow of milk, assuring her it was my confident opinion that in two or three weeks she would have milk, and a sufficient quantity, at least her usual supply.

She did so, and in about two weeks the secretion of milk was reproduced. She continued to nurse her child for more than a year, producing her accustomed quantity of milk.

Case II. Mrs. N. D., aged about twenty-five years, was confined in December, 1841. Nothing worthy of note transpired during her confinement and recovery. In April following her child weaned itself, in consequence of a sore mouth. Her milk soon entirely disappeared. In July following I was called to see her child, which was suffering from an attack of cholera infantum. Having lost several children about that time from this disease, I expressed my regret that the child was deprived of the benefits of the breast, adding, that in my opinion its chances of recovery were diminished in consequence.

The mother was informed of the course I had advised in other cases where it was desirable to reproduce the secretion, and of the results. On my visit the succeeding day, she informed me that she had applied the child to the breast, and that it nursed and seemed pleased and more quiet; but she was not aware that any milk was obtained, or that she had any for it. I advised her to persevere in the application of the child to the breasts, which she did, and the child recovered, and in the course of a week or ten days obtained a full supply of nutriment from the breasts.

The mother continued to nurse for months, with as full and perfect a secretion of milk as though no interruption in the secretion had occurred.

The following case I report as having an important practical bearing on the treatment and disposal of a class of cases which occur in our community at the present day, to cure which or otherwise dispose of satisfactorily to the physician, is often found difficult.

Case III. Mrs. O. H. H., aged about twenty-one years, of feeble constitution and nervo-lymphatic temperament, was confined in July, 1847. Previous to her accouchement she was troubled with chronic aphtha, red canker, or with that condition of the system which is well known as "sore mouth attendant on pregnancy and lactation." Nothing unusual occurred at the time of delivery. No considerable loss of blood was sustained. As in similar cases, there was a remission of diarrhœa and sore mouth for a few days after accouchement, giving rise to a hope that, being relieved from the condition of pregnancy, she would recover the powers of digestion and the assimilation of nutriment so as to enable the system to sustain the calls upon it consequent to lactation. But in the course of ten or twelve days after accouchement the sore mouth and diarrhœa returned with increased violence, producing great debility. The secretion of milk was copious. Her pulse 120; the tongue flabby; there were frequent dejections of yellowish water, the face and extremities bloated, &c. Fearing the worst results for my patient, I advised the immediate removal of the child from the breasts of the mother to those of a wet nurse, at the same time informing the parents that on the recovery of the mother she could at pleasure reapply the child to the breasts and have a full supply of milk, and be enabled to perform all the duties and functions of a mother for an indefinite period of time.

The child was given in charge of a wet nurse, the milk gradually disappeared and the patient recovered under the use of tonic remedies and a generous diet. Between two and three months after this the mother called on me, having the appearance of restored health, and inquired if she might now take her child home with a hope of realizing my former assurances that she would be able to reproduce her milk. I assured her there was no doubt in relation to such a result, and her ability for the future to nurse her child. She took the child, applied it to the breasts, and in the course of two weeks had a good supply of milk.

I met her some nine months after, when she informed me she was happy in the enjoyment of good health, and, to use her words, she "had as good a breast of milk as if she had never dried it up."

